# Differential Raman backscattering cross sections of black carbon nanoparticles

**DOI:** 10.1038/s41598-017-17300-6

**Published:** 2017-12-07

**Authors:** Kim Cuong Le, Christophe Lefumeux, Thomas Pino

**Affiliations:** Institut des Sciences Moléculaires d’Orsay, CNRS, Univ Paris Sud, Université Paris-Saclay, F-91405 Orsay, France

## Abstract

We report the measurements of the differential Raman backscattering cross sections for several carbonaceous ultrafine particles of environmental relevances. These were obtained by dispersing the target particles in liquid water which was used as the internal standard reference. The optical collection was performed in a configuration to ensure a detection as close as possible to the backward direction. These are the first cross sections on black carbon-type particles although Raman spectroscopy is widely used in Carbon science. The high values of the cross sections, few 10^−28^ cm^2^.sr^−1^.atom^−1^, reflect resonance effects that take advantages of the disordered polyaromatic structures. Because they were measured in conditions intended to mimic the aerosol phase, these measurements provide a crucial step to move toward quantitative Raman spectroscopy and enable development of dedicated teledetection of black carbon in the atmosphere and in combustion chambers.

## Introduction

Natural carbonaceous matter, found on Earth as well as in space, plays an effective bridge-building role between astrophysics and geophysics^1^. Crystalline to amorphous forms of carbon find many technological applications, with a high potential for the new carbon nanomaterials^2^. In addition, combustion of carbon-based fuels, including fossil fuels, biomass and biofuels, is still the main source of energy utilized by anthropic activities and dominates carbon-based materials production. Every year, about 10^7^ tons of black carbon (BC, atmospheric light absorbing particles that embrace soot) are emitted world wide^3^. As a consequence, BC occurred in 62% of the total mass of the particles in the polluted air in Mexico^4^. Southeast Asia is one of the main hot spot of BC emission as it combines massive biomass burning events and a growing use of fossil fuels^5^. BC are one of the most efficient climate radiative forcer^3,6^. Secondary effects are also important, such as decreasing the albedo of snow and ice^7–9^. The net impact of BC on climate depends on many factors including the compounds, the presence of co-emitted pollutants, atmospheric lifetime, altitude, aging and mixing processes in the atmosphere, in which the aging of particles plays a key role in the estimation of both the direct and indirect climate effects^10–12^. In addition to climate effects, there are numerous epidemiological studies establishing associations between exposure to particulate pollution and increased morbidity and mortality for respiratory and cardiovascular diseases^13–17^. It is thus of the uttermost importance to progress on the knowledge of their optical properties in order to better evaluate their climate impact. In addition, to help mitigating their emission, it is mandatory to continue the development of new tools to monitor the BC in the atmosphere or soot in combustion chamber.

Raman spectroscopy is a powerful tool for structural investigation of the carbon-based materials because it is sensitive not only to the crystalline structures but also to the molecular structures in amorphous matter^18,19^ and is already widely applied in environmental research^20^ or combustion^21,22^. The main features in Raman spectra of polyaromatic carbonaceous materials are the so-called D and G peaks, highlighted by the pioneering work by Tuinstra and Koenig in 1970^23^. The G peak corresponds to the bond stretching of all pairs of sp^2^ sites in hexagonal aromatic rings. The D peak is due to the breathing modes of sp^2^ sites in rings and its activation requires defects^23^. However, despite the large amount of experimental data available in the literature concerning the Raman spectra of carbonaceous materials^24,25^, most of the studies focused on the relative intensities and shapes of the Raman peaks^18,19,26^ but not on the absolute intensities. In fact, spontaneous Raman spectroscopy is rarely used as a quantitative spectroscopy in Carbon science because making the Raman spectroscopy quantitative for absorbing species remains a challenge.

The present work provides experimental measures of the differential Raman backscattering cross sections (DRCS) of several natural and artificial carbon-based fine particles, together with the development of a practical method to quantify DRCS of other nanoparticles. The samples were chosen to provide a representative set of black carbon particles of atmospheric relevance.

## Results

### Raman spectra of black carbon particles in liquid water

**Figure 1:**
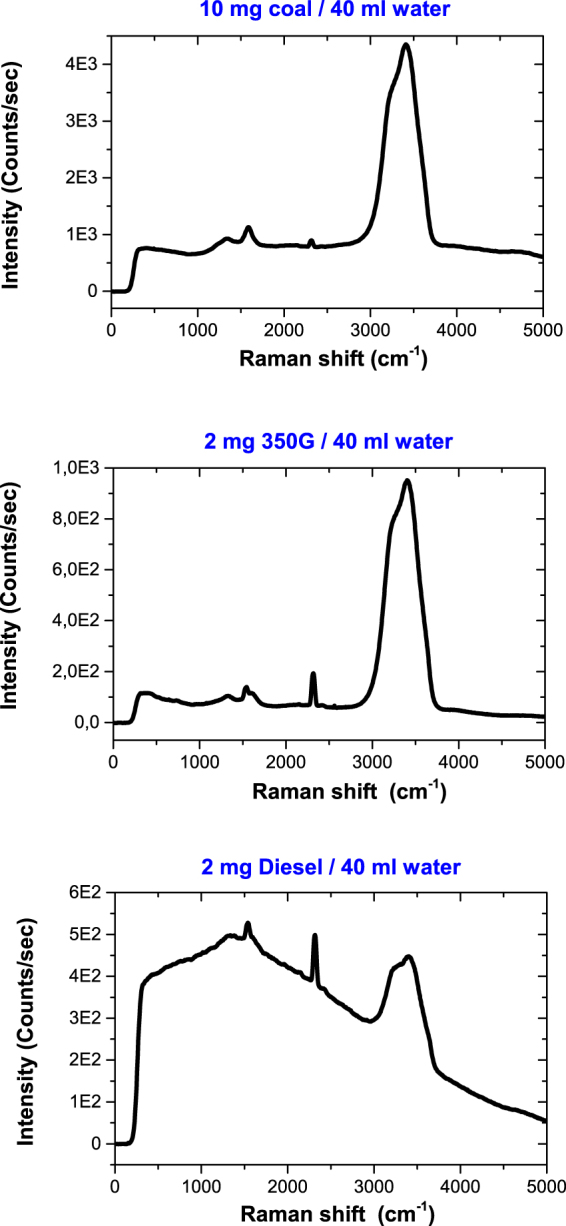
Spectra from the dispersion of several BC particles in liquid water, measured close to the backward direction, during illumination of the sample by a cw laser at 532 nm. Experimental conditions were similar except the concentrations. The horizontal scales are the Raman shift relatively to the incident photon energy. The concentrations are indicated. The dominant band at about 3500 cm^−1^ is the Raman band of liquid water involving the O-H stretching mode.

The Raman spectra of the BC particles in liquid water are shown in Fig. 1. The superimposition of the typical G and D Raman bands at about 1600 and 1350 cm^−1^, respectively^18,19,27^, with those of liquid water at about 1640 and 3500 cm^−1^ ^28,29^ can be seen. The diesel soot spectrum exhibits a strong background but the Raman bands remain observable. The background is due to fluorescence, probably originating from the polycyclic aromatic hydrocarbons (PAHs) that are adsorbed on the soot particles. Additional bands can be seen which correspond to the Raman spectra of O_2_ (at 1555 cm^−1^) and N_2_ (at 2331 cm^−1^) from the atmospheric column along the line of sight. It should be noted that the absolute intensities of the N_2_ and O_2_ Raman bands are found almost constant in Fig. 1, only the BC and water Raman bands relative intensities are found varying.

**Figure 2:**
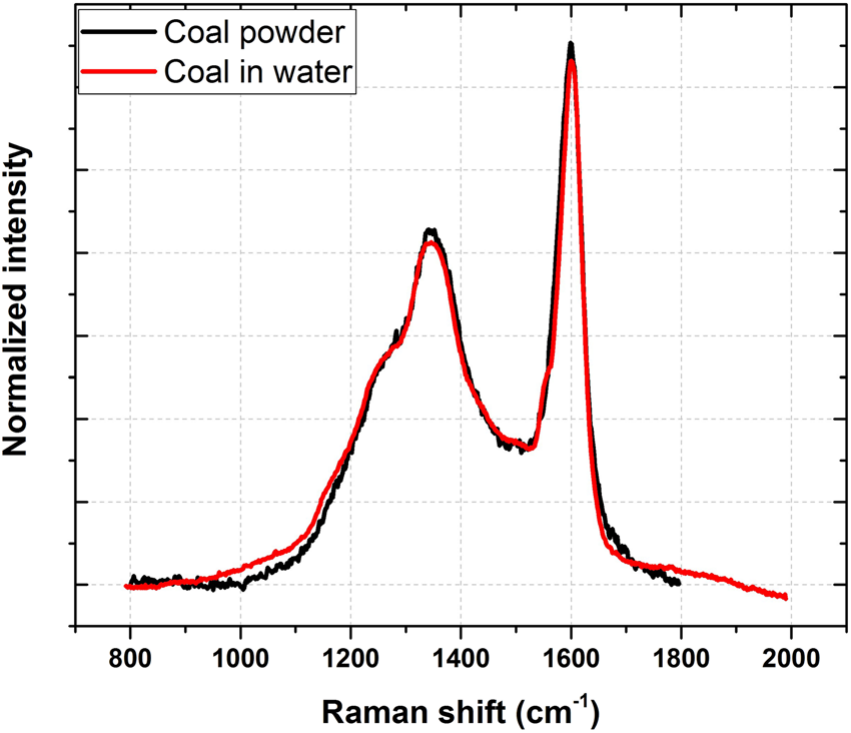
Coal particles Raman spectrum in the form of powder superimposed to that of the coal particles Raman spectrum dispersed in liquid water. The underlying continuum were subtracted by a simple linear background.

In Fig. 2, the coal particles Raman spectrum in the form of powder is superimposed to that of the coal particles dispersed in liquid water. The overlap shows that the two spectra are very similar and confirms that the solvent has no measurable effects on the Raman bands of the coal particles. The slight differences are due to the subtraction of the background and of the liquid water Raman band lying at 1640 cm^−1^ ^28^.

### Measurements of the Raman cross sections

**Figure 3:**
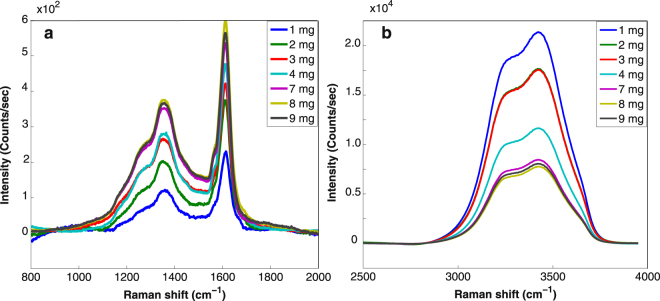
Panel (a) Evolution of the coal particles Raman bands as a function of concentration. Panel (b) Evolution of the liquid water Raman band (stretching mode) as a function of concentration. Contribution of the water bending mode was subtracted, as contrained by the pure water Raman spectrum. Background was subtracted by a simple linear shape.

**Figure 4:**
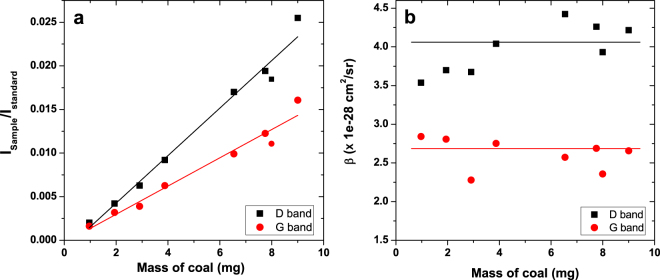
Panel (a) Intensity ratios of the Raman bands as a function of the coal particles concentrations. The slopes are found to be 0.0027 and 0.0016 for the D and G bands, respectively. Panel (b) DCRS for the G (red lines and points) and D (black line and points) bands of coal particles deduced at all concentrations, together with the average value.

After systematic subtraction of the underlying background and of the contribution of the bending mode of water, all obtained Raman spectra of coal and water stretching bands of the mixtures at different coal concentrations are plotted in Fig. 3. Note that the O-H stretching band is much more intense than the D and G bands. It can be seen that the higher the concentration of the coal particles is, the higher the Raman signal of coal is. An opposite trend in the O-H stretching band is concomitantly observed because the probe volume decreases with the increase of the optical density (following the increase of coal concentration) of the solution. The D band intensity was taken by summing the photoelectron counts from 1000 to 1500 cm^−1^ and for the G band intensity from 1500 to 1700 cm^−1^. For the stretching mode intensity of liquid water, no other feature contaminates its spectral range and integration was performed all over the band. The ratios between the observed Raman signals of the D and G bands and the O-H stretching band of the liquid water are shown in Fig. 4 as a function of coal particles concentrations. The experimental ratios are following linearly the coal concentrations **(**Fig. 4a **)** as expected for a pure dependence on the mass ratios even if the probe volume is changing due to the dependence of optical extinction of the solution with concentration. It confirms that the DRCS of the D and G bands can be evaluated using the liquid water band as an internal standard. The DRCS for the stretching mode of water is taken to be β* = 5.74 10^−30^ cm^2^.sr^−1^.molecule^−1^ ^28,30^. From equation 5, the DRCS of D and G peaks are found to be 4.1 ± 1.1 × 10^−28^ cm^2^.sr^−1^.atom^−1^ and 2.7 ± 0.6 × 10^−28^ cm^2^.sr^−1^.atom^−1^, respectively, as deduced from the average value of the DRCS deduced at each concentration (Fig. 4b). Note that the carbon content is 92% of the mass of coal and this value was taken into account.

**Figure 5:**
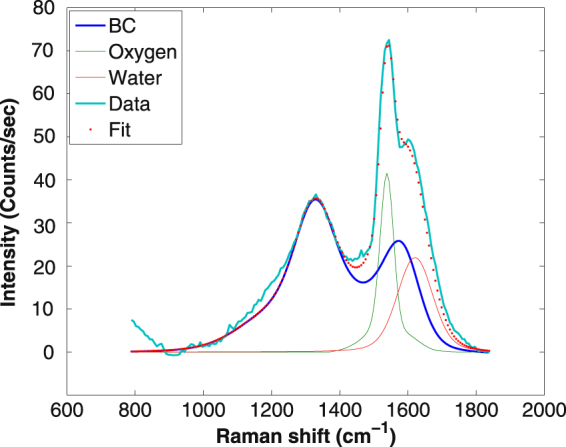
Deconvolution of the Raman spectrum of Ensaco 350G carbon black nanoparticles dispersed in liquid water (about 2 mg in 40 ml of water). The bright blue line is the experimentally observed Raman spectrum; The blue solid line is the Raman spectrum of the CB in form of powder; The green solid line is the scaled Raman signal of O_2_; The red solid line is the fitted bending Raman band of water; The red dot line is the fitted spectrum from the three components contributing to the experimental data.

The Raman spectra of CB Ensaco 350G dispersed in liquid water were more complicated to analyze than that of coal particles. While the D peak appeared clearly (Fig. 5) with no spectral contamination, the G peak was found to overlap with the atmospheric O_2_ band and the bending mode of water. In addition subtraction of the bending mode contamination could not be performed using the pure water bending/stretching ratio: the measured liquid water bending mode signal was evidently weaker than expected. It traces an interaction of the solvent with the nanoparticles, their nanometer size shape (≈30 nm diameter^31^) offering a much higher solvation surface than the coal microparticles (average diameter about 1 *μ*m). A comparison of the shapes of the water stretching band at different concentrations shows that no spectral changes are observed. It underlines a weak effect for this mode. Therefore the data treatment assumed that the water stretching mode cross section was unchanged.

**Figure 6:**
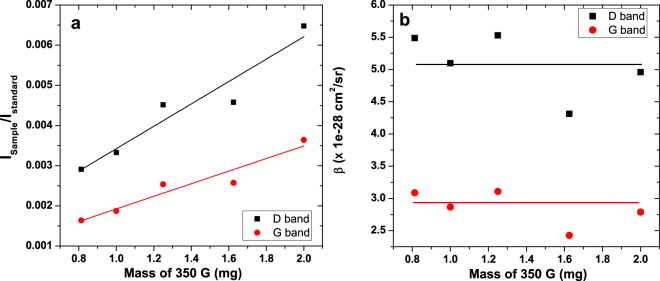
Panel (a) Intensity ratios of the Raman bands as a function of the CB nanoparticles concentrations. The slopes are found to be 0.0028 and 0.0016 for the D and G bands, respectively. Panel (b) DCRS for the G (red lines and points) and D (black line and points) bands of the CB nanoparticles deduced at all concentrations, together with the average value.

To fit the experimental spectra, we summed all components using the CB powder Raman spectrum and the O_2_/N_2_ Raman signals to determine the strength of the O_2_ Raman band independently. The shape of the water bending mode was taken from the pure liquid water Raman data. The result of the fit is shown in Fig. 5. We carried out this analysis for several CB concentrations. The ratios of the observed Raman signals of the D, G and O-H stretching bands are plotted in Fig. 6a and exhibit the expected linear trend. The deduced DRCS for all concentrations are shown in Fig. 6b using equation 5. It should be noted that due to the higher optical density of these solutions, the range of concentration was reduced to 0.8–2 mg in 40 ml water, i.e. a smaller range as compared to that of the coal particles (Fig. 4). We obtained the average DRCS for the D band 5.1 ± 1.2 × 10^−28^ cm^2^.sr^−1^.atom^−1^ and for the G band 2.9 ± 0.8 × 10^−28^ cm^2^.sr^−1^.atom^−1^.

**Figure 7:**
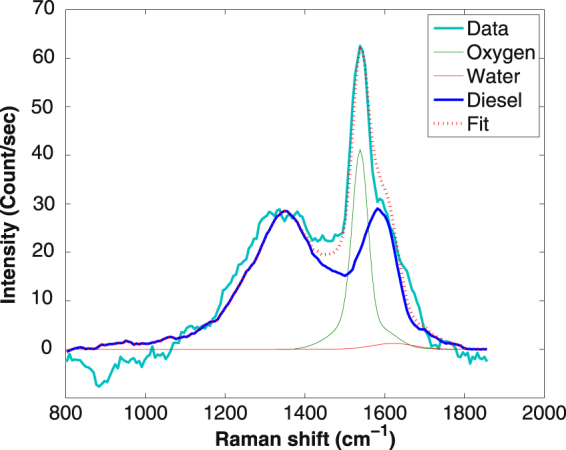
Deconvolution of the Raman spectrum of 9 mg of soot SRM 2975 (NIST) in 40 ml of water. The bright blue line is the experimentally observed Raman spectrum; The blue solid line is Raman spectrum of sample in form of powder; The green solid line is Raman signal of O2 and water; the red denoted line is the sum of the 3 components.

For the diesel soot, the measurements were done at two different concentrations: 9 mg and 5.4 mg of diesel soot in 40 ml water. The spectral contamination in the range of the G band is found similar to that the CB Ensaco 350G and the same fit was applied to extract the G band intensity (Fig. 7). In that case, the signal to noise ratio was even worse than that obtained for the CB Ensaco 350G, precluding measurements at lower concentrations. The average DRCS of the G and D bands are 8.8 ± 3 × 10^−28^ cm^2^.sr^−1^.atom^−1^ and 11 ± 4 × 10^−28^ cm^2^.sr^−1^.atom^−1^, respectively.

## Discussion

The DCRS values are reported in the Table 1. There is no large difference between the DRCS of the three samples, which are on the order of few 10^−28^ cm^2^.sr^−1^.atom^−1^. It should be noted that these are effective cross sections. Actually the measurements were scaled with respect to their mass (number of carbon atoms) although the granularity may have an influence on the scattering properties. For the coal particles, sizes were about 1 *μ*m as revealed by optical microscopy on dried samples. The two other samples were composed of nanoparticles of about few tens of nm diameter, generally forming aggregates of up to few hundreds of nm size. It should be noted that the exact size distribution while being dispersed in liquid water under ultrasonication in the cleaning unit was not controlled. Thus, although the granularities were different for all samples, the linear dependence with concentration for the coal particles and the CB Ensaco 350G provide strong support for our values per carbon atoms.

**Table 1:** Differential Raman cross sections for carbon-based materials together with those of some molecules. a: cross sections for molecules are usually given per molecule, here they were divided by the number of carbon atoms composing the molecules for direct comparison.

Sample	Laser excitation *λ* (nm)	Raman shift (cm^−1^)	DRCS (10^−30^ cm^2^.sr^−1^.atom^−1^)	Reference
N_2_ gas	515.4	2331	0.43	Ref.^47^
O_2_ gas	514.5	1555	0.58	Ref.^47^
Benzene liquid^*a*^	514	992	5.42	Ref.^38^
Toluene liquid^*a*^	488	1002	2.6	Ref.^38^
C_60_^*a*^	752.5	1469	0.348	Ref.^35^
Graphene	514.5	1580	227	Ref.^48^
Graphite	514.5	1585	≈700	Ref.^32^
Nanographite (L = 20 nm)	514.5	1350	20	Ref.^33^
		1580	20	Ref.^33^
Nanographite (L = 35 nm)	514.5	1350	13	Ref.^33^
		1580	28	Ref.^33^
Nanographite (L = 65 nm)	514.5	1350	7	Ref.^33^
		1580	30	Ref.^33^
*Ia natural diamond*	514.5	1332	27	Ref.^34^
Coal microparticles	532	1357	410	Our work
		1610	270	Our work
Ensaco 350G	532	1344	510	Our work
		1596	290	Our work
Diesel soot (SRM 2975, NIST)	532	1348	1100	Our work
		1582	880	Our work

The DRCS for Raman modes of various types of carbonaceous materials have been measured for only few carbonaceous materials that are graphite^32,33^, diamond^34^, C_60_^35^ and carbon nanotubes^36^. In the latter case, the values were not reported in Table 1 because it could not be given per carbon atom. Our study shows that the DRCS of our samples are approximately 100 times larger than that of the breathing mode of liquid benzene. It is also higher than that of diamond. The DRCS of coal, CB and diesel soot are found comparable to the graphite values but higher than those of the nano-graphites. For those bulky materials, the variations cannot involve the particle shapes scattering properties. The reason for the large cross sections is probably the appearance of resonance Raman effect in our samples leading to the enhancement of Raman signals^37^ and was shown to follow a polyaromatic unit size dependence^33^. The FWHMs of the D and G bands of our samples are much larger than those of the nano-graphites in Cançado *et al*.^33^. The FWHMs of their Raman bands range from 10 to 50 cm^−1^ while in our BC particles, they are about several tens to 200 cm^−1^. It shows that the polyaromatic unit size *La* of our samples are much smaller than those of the nano-graphites, ranging from 20 to 65 nm^33^. Effectively diesel soot have *La* of about 2 nm^17^, carbon black around 1–2 nm^31^ and the anthracite coal exhibits an average *La* of about 2 nm as well (measured by high resolution transmission electron microscopy, not shown). It suggests that the resonance effects should be similar for the BC samples, as observed. In order to progress further and explain the high values, the DCRS would have to be measured at several wavelengths in order to probe the resonant effects from the near-infrared up to the UV.

An important outcome of this investigation is that it reveals that the DRCS of BC are large. These are found close to that of graphite and nearly 3 orders of magnitude higher that of gaseous molecules^38^ (toluene, benzene…) or silicates^39^ commonly at the focus of atmospheric research. We believe our determination of the DRCS will contribute to the development of quantitative teledetection of BC emission in the atmosphere, for instances by extending Raman-Lidar capabilities recently developed for silicate aerosols^39–41^ or in combustion chambers by complementing the laser induced incandescence technique^42,43^ recently applied in atmospheric measurements^44^. The main drawback relies on the observed variations of the DRCS among the present set of reference BC up to a factor of 3. It is in line with the specificity of the BC family of aerosol whose optical properties are spanning a large range of values^45^. Additional work has to be performed in order to determine the best DCRS values to be used when monitoring complex mixtures of BC. It is noteworthy that the measure of the Raman G and D bands characteristics provide very valuable information for speciation as well. Overall, *μ*g/m^3^ of BC concentration appear as a plausible target for Raman teledetection.

## Methods

### Calibration method for the Determination of the cross section

The DCRS *β* (in cm^2^.sr^−1^.atom^−1^) for a vibrational mode is defined as the number of inelastically scattered photons per unit time and per unit solid angle divided by the incident number of photons per unit time per unit area. It relates to the observed signal *S* (in photoelectron)^38^:

1 $$S=({P}_{D}\mathrm{.}\beta \mathrm{.}D\mathrm{.}K\mathrm{).(}{A}_{D}\mathrm{.}{O}_{D}\mathrm{.}T\mathrm{.}Q\mathrm{).}t$$

where *P*_*D*_ (in photons.s^−1^.cm^−2^) is the photon irradiance, *D* (in molecules.cm^−3^) the density of scatterers, *K* the constant of geometry (unit less), *A*_*D*_ (in cm^2^) the sample area monitored by the spectrometer, *O*_*D*_ (in sr) the collection solid angle of the spectrometer, at the sample, T the transmission of the spectrometer and collection optics (unit less), *Q* the quantum efficiency of the detector (in e^−^/photon), and t is the observation time (in seconds). The first composition is the characteristic intensity defined as the total Raman scattering divided by the area of scattering volume. The second is a general instrumental component containing the collection optics and spectrograph. The DRCS in the equation 1 is difficult to use directly because it depends on a large number of variables. Thus we simplified the measurements by using internal calibration. It consists in normalizing the observed signal of the sample to that of the co-mixed standard reference (indicated by * in the following equations). If the sample and the standard substance, whose Raman cross section is known, are measured simultaneously, they have the same photon irradiance, the sample area monitored by the spectrometer, the same collection solid angle of the spectrometer and the same acquisition time. In this case, the ratio *S*/*S* ^*^ is given by:

2 $$S/{S}^{\ast }=(\beta \mathrm{.}D\mathrm{.}T\mathrm{.}Q)/({\beta }^{\ast }\mathrm{.}{D}^{\ast }\mathrm{.}{T}^{\ast }\mathrm{.}{Q}^{\ast })$$

The observed signals in photoelectrons are proportional to the number of digital units “counts” I of CCD camera. So, the equation 2 is re-written:

3 $$I/{I}^{\ast }=(\beta \mathrm{.}D\mathrm{.}T\mathrm{.}Q)/({\beta }^{\ast }\mathrm{.}{D}^{\ast }\mathrm{.}{T}^{\ast }\mathrm{.}{Q}^{\ast })$$

The composition of the transmission T and the efficiency Q depends on the type of detector, grating, and optics used. Those parameters were measured with a calibration source. We used a blackbody calibration source BB4-A (Omega Engineering Inc.), whose source temperature is adjustable over a range of 100 to 982 Celsius degrees. The black body was installed at the sample position. The ratio between the Raman peaks of the samples and of the standard reference after being corrected with the spectral response then simplifies:

4 $${I}_{corrected}/{I}_{correcte{d}^{\ast }}=(\beta \mathrm{.}D)/({\beta }^{\ast }\mathrm{.}{D}^{\ast })$$

From the equation 4, the DRCS of the sample, *β*, is determined by:

5 $$\beta =({I}_{corrected}\mathrm{.}{D}^{\ast })/({I}_{correcte{d}^{\ast }}.D\mathrm{).}{\beta }^{\ast }$$

This is the final equation used to deduce the DRCS of the samples. In our experiment, the dimension of D* is water molecule.cm^−3^ while that of D is carbon atom.cm^−3^.

A crucial advantage of our method is that it permits the measurement of differential cross sections of particles using internal calibration. It allows to avoid the difficulty inherent to the case of absorbing material while the references are usually transparent^38^.

### Samples preparation

The samples were anthracite coal ultrafine particles from a coal sample originating from a mine in Vietnam and milled in an Agate mortar, an industrial carbon black (CB) Ensaco 350G (Timcal Graphite & Carbon) and the soot produced from a diesel engine (SRM2975, NIST) called diesel soot in this paper. The diesel particulate material used to prepare SRM 2975 was obtained from M.E. Wright of the Donaldson Company, Inc., Minneapolis, MN. The material was collected from a filtering system designed specifically for diesel-powered forklifts^46^.

For the measurements, each sample was mixed in pure water at high concentrations. The beakers were installed in the water tank of an ultrasonic cleaning unit (Elmasonic) operated at 37 kHz frequency and at 40W. After a half an hour, the majority of their masses were dissolved or suspended in the solvent, the rest was found floated on the surface and was removed and weighted for correction. Then dilution was applied to prepare solutions with targeted concentrations. Note that their concentrations were ensured transparent enough for transmission of the laser. During the acquisition, the samples were kept in the ultrasonic cleaning unit to prevent from sedimentation on the bottom of the beaker.

It should be noted that we tried using other solvents as internal standard: cyclohexane and ethanol. However, as most organic solvents, their Raman spectra exhibits several strong bands overlapping with the D and G bands of the BC samples and hiding them. Although the bending mode of water at 1640 cm^−1^ is close to G peak, its DRCS is weak enough to enable subtraction. Thus water appeared as the best solvent when using ultrasonication to prepare the mixtures and during the measurements.

### Experimental set up

**Figure 8:**
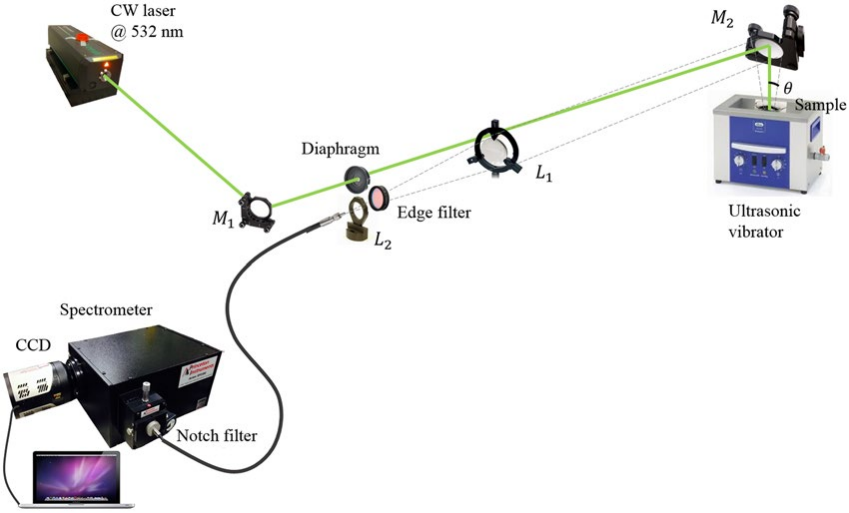
Scheme of the experimental setup. M1 is a laser mirror at 532 nm, M2 a broadband dielectric mirror covering the visible range, L1 and L2 two focus lengths.

In our experimental setup (Fig. 8), the excitation source was a continuous laser firing at 532 nm (Verdi, Coherent). The laser was illuminating the sample from the top using a broadband dielectric laser mirror to avoid fluorescence from the glass of the beaker. The scattered light was collected by a lens system positioned about 1.5 m from the target, filtered by a 532 nm Razor Edge filter before entering a round-to-linear fiber bundle. This filter blocks the radiations from 430 to 539 nm with an optical density (OD) greater than 6 and transmits up to 90% from 539 to 1200 nm. The fiber bundle contains 19 fibers arranged in a line configuration at one end and in a circular configuration at the other end. Each fiber has a core diameter of 200 *μm*. The linear end coupled with the slit of a spectrometer equipped with a CCD camera (model PIXIS 400, Princeton Inst.). The spectrometer was a 0.3 m focal length (Acton SP2300 spectrometer, Princeton Inst.) equipped with a 1200 lines grating blazed at 500 nm. A notch filter at 532 nm was inserted in front of the slit of the spectrometer to further reject the elastic scattering. This StopLine notch filter (Semrock) has OD greater than 6 blocking of wavelength at 532 nm and about 90% transmission from 551 to 1600 nm. The optical fibers are not polarization maintaining therefore all measurements were unpolarized. Typical laser power were about 0.2W and acquisition time for one spectrum was set to about 20 minutes. Each measurement was performed several times to ensure reproducibility and check that the concentrations were constant during the measurements.

## References

[1] Ehrenfreund P, Charnley S (2000). Organic molecules in the interstellar medium, comets, and meteorites: A voyage from dark clouds to the early earth. Ann. Rev. Astrophys. Astron..

[2] Delhaes, P. *Carbon-based Solids and Materials* (John Wiley& Sons, Ltd, 2011).

[3] Bond TC (2013). Bounding the role of black carbon in the climate system: A scientific assessment. Journal of geophysical research.

[4] Buseck PR, Adachi K, Gelencsér A, Tompa É, Pósfai M (2014). Ns-Soot: A Material-Based Term for Strongly Light-Absorbing Carbonaceous Particles. Aerosol Science and Technology.

[5] Reid JS (2013). Observing and understanding the Southeast Asian aerosol system by remote sensing: An initial review and analysis for the Seven Southeast Asian Studies (7SEAS) program. Atmospheric Research.

[6] Ramanathan V, Carmichael G (2008). Global and regional climate changes due to black carbon. Nature Geoscience.

[7] Schmale, J. *et al*. Modulation of snow reflectance and snowmelt from Central Asian glaciers by anthropogenic black carbon. *Scientific Reports* **7**, 10.1038/srep40501 (2017). 10.1038/srep40501 PMC5228185 28079148

[8] Qian Y (2015). Light-absorbing particles in snow and ice: Measurement and modeling of climatic and hydrological impact. Advances in Atmospheric Sciences.

[9] Schwarz, J. P., Gao, R. S., Perring, A. E., Spackman, J. R. & Fahey, D. W. Black carbon aerosol size in snow. *Scientific Reports* **3**, 10.1038/srep01356 (2013). 10.1038/srep01356 PMC3584901 23449011

[10] Cakmak S, Dales RE, Angelica Rubio M, Blanco Vidal C (2011). The risk of dying on days of higher air pollution among the socially disadvantaged elderly. Environmental Research.

[11] Moffet RC, Prather KA (2009). *In-situ* measurements of the mixing state and optical properties of soot with implications for radiative forcing estimates. Proc. Nat. Acad. Sciences.

[12] Riemer N, Vogel H, Vogel B (2004). Soot aging time scales in polluted regions during day and night. Atmospheric Chemistry and Physics.

[13] Val S (2013). Physico-chemical characterization of African urban aerosols (Bamako in Mali and Dakar in Senegal) and their toxic effects in human bronchial epithelial cells: description of a worrying situation. Particle and fibre toxicology.

[14] West JJ (2016). What We Breathe Impacts Our Health: Improving Understanding of the Link between Air Pollution and Health. Environmental Science & Technology.

[15] Crippa, P. *et al*. Population exposure to hazardous air quality due to the 2015 fires in Equatorial Asia. *Scientific Reports* **6**, 10.1038/srep37074 (2016). 10.1038/srep37074 PMC5111049 27848989

[16] Poeschl U, Shiraiwa M (2015). Multiphase Chemistry at the Atmosphere-Biosphere Interface Influencing Climate and Public Health in the Anthropocene. Chemical Reviews.

[17] Kocbach A (2006). Physicochemical characterisation of combustion particles from vehicle exhaust and residential wood smoke. Particle and Fibre Toxicology.

[18] Ferrari A, Robertson J (2000). Interpretation of Raman spectra of disordered and amorphous carbon. Physical Review B.

[19] Sadezky A, Muckenhuber H, Grothe H, Niessner R, Poeschl U (2005). Raman microspectroscopy of soot and related carbonaceous materials: Spectral analysis and structural information. Carbon.

[20] Ferrugiari A, Tommasini M, Zerbi G (2015). Raman spectroscopy of carbonaceous particles of environmental interest. Journal of Raman Spectroscopy.

[21] Russo C, Ciajolo A (2015). Effect of the flame environment on soot nanostructure inferred by Raman spectroscopy at different excitation wavelengths. Combustion and Flame.

[22] Commodo M, D’Anna A, De Falco G, Larciprete R, Minutolo P (2017). Illuminating the earliest stages of the soot formation by photoemission and Raman spectroscopy. Comb. & Flame.

[23] Tuinstra F, Koenig JL (1970). Characterization of Graphite Fiber Surfaces with Raman Spectroscopy. Journal of Composite Materials.

[24] Pimenta M (2007). Studying disorder in graphite-based systems by Raman spectroscopy. Physical Chemistry Chemical Physics.

[25] Ferrari AC, Basko DM (2013). Raman spectroscopy as a versatile tool for studying the properties of graphene. Nature Nanotechnology.

[26] Casiraghi C, Ferrari AC, Robertson J (2005). Raman spectroscopy of hydrogenated amorphous carbons. Physical Review B.

[27] Lespade P, Marchand A, Couzi M, Cruege F (1984). Caracterisation de materiaux carbones par microspectrometrie Raman. Carbon.

[28] Bray A, Chapman R, Plakhotnik T (2013). Accurate measurements of the Raman scattering coefficient and the depolarization ratio in liquid water. Applied optics.

[29] Carey D, Korenowski G (1998). Measurement of the Raman spectrum of liquid water. Journal of Chemical Physics.

[30] Plakhotnik T, Reichardt J (2017). Accurate absolute measurements of the Raman backscattering differential cross-section of water and ice and its dependence on the temperature and excitation wavelength. J. Quant. Spec. Rad. Trans..

[31] Pawlyta M, Rouzaud J-N, Duber S (2015). Raman microspectroscopy characterization of carbon blacks: Spectral analysis and structural information. Carbon.

[32] Wada N, Solin SA (1981). Raman efficiency measurements of graphite. Physica B+C.

[33] Cançado LG, Jorio A, Pimenta MA (2007). Measuring the absolute Raman cross section of nanographites as a function of laser energy and crystallite size. Physical Review B.

[34] Aggarwal RL (2012). Measurement of the absolute Raman cross section of the optical phonons in type Ia natural diamond. Solid State Communications.

[35] Lorentzen JD, Guha S, Menéndez J, Giannozzi P, Baroni S (1997). Raman cross section for the pentagonal-pinch mode in buckminsterfullerene C60. Chemical Physics Letters.

[36] Bohn JE (2010). Estimating the Raman Cross Sections of Single Carbon Nanotubes. ACS Nano.

[37] Ferrari AC, Robertson J (2001). Resonant Raman spectroscopy of disordered, amorphous, and diamondlike carbon. Physical Review B.

[38] Mccreery, R. L. *Photometric Standards for Raman Spectroscopy* (John Wiley& Sons, Ltd, 2006).

[39] Tatarov B, Sugimoto N (2005). Estimation of quartz concentration in the tropospheric mineral aerosols using combined Raman and high-spectral-resolution lidars. Optics letters.

[40] Mueller, D. *et al*. Mineral quartz concentration measurements of mixed mineral dust/urban haze pollution plumes over Korea with multiwavelength aerosol Raman-quartz lidar. *Geophysical Research Letters* **37**10.1029/2010GL044633.(2010).

[41] Sugimoto N, Zhongwei H (2014). Lidar methods for observing mineral dust. Journal of Meteorological Research.

[42] Schulz C (2006). Laser-induced incandescence: recent trends and current questions. Applied Physics B-Lasers and Optics.

[43] Michelsen HA (2017). Probing soot formation, chemical and physical evolution, and oxidation: A review of *in situ* diagnostic techniques and needs. Proc. Comb. Inst..

[44] Miffre A, Anselmo C, Geffroy S, Frejafon E, Rairoux P (2015). Lidar remote sensing of laser-induced incandescence on light absorbing particles in the atmosphere. Optics Express.

[45] Bond TC, Bergstrom RW (2006). Light Absorption by Carbonaceous Particles: An Investigative Review. Aerosol Science and Technology.

[46] Wright, M., Klein, A. & Stesniak, E. A Diesel Exhaust Filter System for Industrial Diesel Forklifts. *SAE Technical Paper* **91185283** (1991).

[47] Schrotter, H. W. & Klackner, H. W. *Raman scattering cross sections in gases and liquids* (Springer- Verlag Berlin Heidelberg, 1979).

[48] Klar P (2013). Raman scattering efficiency of graphene. Physical Review B - Condensed Matter and Materials Physics.

